# Telehealth Monitoring for Individuals Experiencing Homelessness During the Early COVID-19 Pandemic: An Innovative Clinical and Medical Education Model

**DOI:** 10.7759/cureus.31528

**Published:** 2022-11-15

**Authors:** Victorien Meray, Aarif Motorwala, Kyle P Sellers, Alex Suarez, Gregory W Schneider

**Affiliations:** 1 Department of Translational Research, Herbert Wertheim College of Medicine, Miami, USA; 2 Department of Humanities, Health, and Society, Herbert Wertheim College of Medicine, Miami, USA; 3 Department of Psychiatry, Massachusetts General Hospital, Boston, USA; 4 Department of Internal Medicine, Newton-Wellesley Hospital, Newton, USA; 5 Department of Humanities, Health, and Society/Family Medicine, Herbert Wertheim College of Medicine, Miami, USA

**Keywords:** covid-19, telehealth, isolation and quarantine, person experiencing homelessness (peh), homelessness

## Abstract

Background: People experiencing homelessness (PEH) are recognized as members of a vulnerable population with significant health and social disparities. Due to the COVID-19 pandemic, these populations are at risk for increased morbidity and mortality. The Herbert Wertheim College of Medicine (HWCOM) of Florida International University (FIU) in collaboration with the Miami-Dade County Homeless Trust presents this case series based on the results of the Telemedicine Homeless Monitoring Project, launched in April 2020.

Methods: Utilizing a faculty-student educational model, medical students at FIU HWCOM called PEH patients residing in isolation hotels on a daily basis to monitor their symptoms using a COVID-19 risk assessment template. Thirty-one PEH patients were followed for the duration of 12 weeks between April 2020 and August 2020. A retrospective chart review was then conducted, and four exemplar patients were chosen, highlighting common themes. Variables in the risk assessment included demographics, comorbidities, past medical history, indications for isolation or quarantine, length of stay, clinical and social needs identified, and qualitative data regarding barriers or successes of the telehealth platform.

Results: Thirty-one patients, between the ages of 20 and 84 and with an average age of 50.74, were followed in the program. There were eight females and 23 males in the study. Four exemplar PEH patients were discussed, highlighting the common themes identified; the lack of basic necessities that PEH face, the burden of chronic medical illnesses, a lack of health literacy, the burden of mental illnesses, and the acute stress caused by COVID-19 itself.

Conclusion: Our research identified numerous characteristics of the homeless population that providers should pay special attention to during the pandemic. The relationship between the Homeless Trust and FIU HWCOM provided medical students with an excellent learning opportunity by letting them participate in clinical care while under lockdown due to the COVID-19 outbreak. Based on the results of the study, we believe that models like this will be useful in the event of a future epidemic.

## Introduction

People experiencing homelessness (PEH) have been recognized as members of a vulnerable population that experiences significant health disparities across a wide range of health conditions [1]. The impact of PEH's socioeconomic vulnerability often leads them to prioritize obtaining basic human needs over seeking health and social care services [2]. Consequently, PEH experience a higher prevalence of infectious diseases, non-communicable diseases, mental illnesses, and substance use disorders [3]. In the context of the COVID-19 pandemic, PEH have faced and continue to face unique challenges in accessing healthcare. The impact of pre-existing health disparities led PEH to experience increased morbidity and mortality during the early phase of the COVID-19 pandemic [4]. Bridging the health and healthcare disparities that PEH face during the pandemic required novel solutions to help address both the sociodynamic impact of homelessness and the need for easily accessible healthcare services for a vulnerable population.

Providing services for homeless individuals who would benefit from quarantine or isolation because of exposure or known coronavirus infection was especially difficult during the early period of the pandemic. Several organizations set up protocols utilizing both remote monitoring and hotel-based quarantine approaches to assist these individuals in quarantine or isolation. The University of California San Francisco Benioff Homelessness and Housing Initiative was an example of such a hotel-based COVID-19 isolation initiative which successfully provided integrated medical and behavioral health assistance to more than 1,000 people outside of a hospital environment in San Francisco [5]. Another novel approach in Atlanta, Georgia, using a telemedicine risk assessment tool to predict the rate of hospitalization in an outpatient population exposed to COVID-19 was found to successfully stratify patients into low-, medium-, and high-risk groups [6]. The transformative effects that COVID-19 has had on our healthcare system are likely to persist beyond the pandemic, and thus it is imperative that medical students be offered training in novel areas to improve access to healthcare amongst PEH (Oral presentation: Mallory C, Alderman M, Abad N, Lobon K, Stumbar S, Schneider G. COVID-19 Homeless Trust Surveillance Educational Project. STFM 2021 Annual Spring Conference; 2021).

The Herbert Wertheim College of Medicine (HWCOM) of Florida International University (FIU) is a community-based medical school with a mission to prepare students to become socially accountable physicians, scientists, and health professionals serving their local communities. Through educational programs such as the Green Family Foundation Neighborhood Health Education Learning Program (NeighborhoodHELP), medical students are given the unique opportunity to follow patients in the community to help address any social or medical needs. The pandemic and subsequent lockdown forced students and faculty members at HWCOM to grapple with an important question: how does the institution maintain everyone's safety while ensuring the school’s educational mission and its community focus?

One response was to create a variety of faculty-student collaborative enterprises to serve various underserved communities in South Florida, including PEH. One such collaboration was the HWCOM Telemedicine Homeless Monitoring Project, launched in April 2020 in conjunction with the Miami-Dade County Homeless Trust. The project provided medical students with educational opportunities and virtual experiences while serving PEH in quarantine or isolation at local hotels in the county because of the COVID-19 pandemic. Students learned about telemedicine, and the pilot program promoted faculty-student collaboration and mentoring. Medical students conducted phone calls and used a COVID-19 risk assessment tool to identify at-risk PEH patients quarantined in hotels, following the faculty-student training model similarly used in other past studies. This case series summarizes the project and showcases the clinical and social needs of the PEH served.

## Materials and methods

Population and sample

Participants in the project included PEH patients that were referred by the Miami-Dade County Homeless Trust as part of a collaboration with the FIU HWCOM Department of Humanities, Health, and Society. The participants were in isolation or quarantine with suspected or confirmed COVID-19 disease at participating hotels in South Florida during the first six months of the pandemic, from March through September. The collaboration with FIU HWCOM continued from April through August 2020. The Miami-Dade County Homeless Trust authorized the use of two hotels close to the Miami International Airport as isolation facilities. The patients in this case series consented to participate in the telemedicine pilot project by signing a Consent for Treatment approved by both the legal team at the Homeless Trust and the General Counsel of FIU. There were no exclusion criteria for participation in the pilot program which included 31 patients. The investigators obtained approval for conducting this retrospective case series from the FIU Institutional Review Board (IRB).

Monitoring protocol

Patients were evaluated and followed by assigned teams of medical students to track patients' symptoms as they were quarantined/isolated in hotels approved by the Miami-Dade County Homeless Trust. A total of six faculty members from HWCOM oversaw between six and eight medical students per team. Teams were divided by patients and each patient was paired with an upper-class and lower-class medical student. Patients were followed for 12 weeks to evaluate and follow up with their symptoms. During this period, each team would call the patient daily using an encrypted phone application to monitor for symptoms. The medical students would be supervised by clinical faculty who were available to provide guidance and participate in a call, as needed.

**Figure 1:**
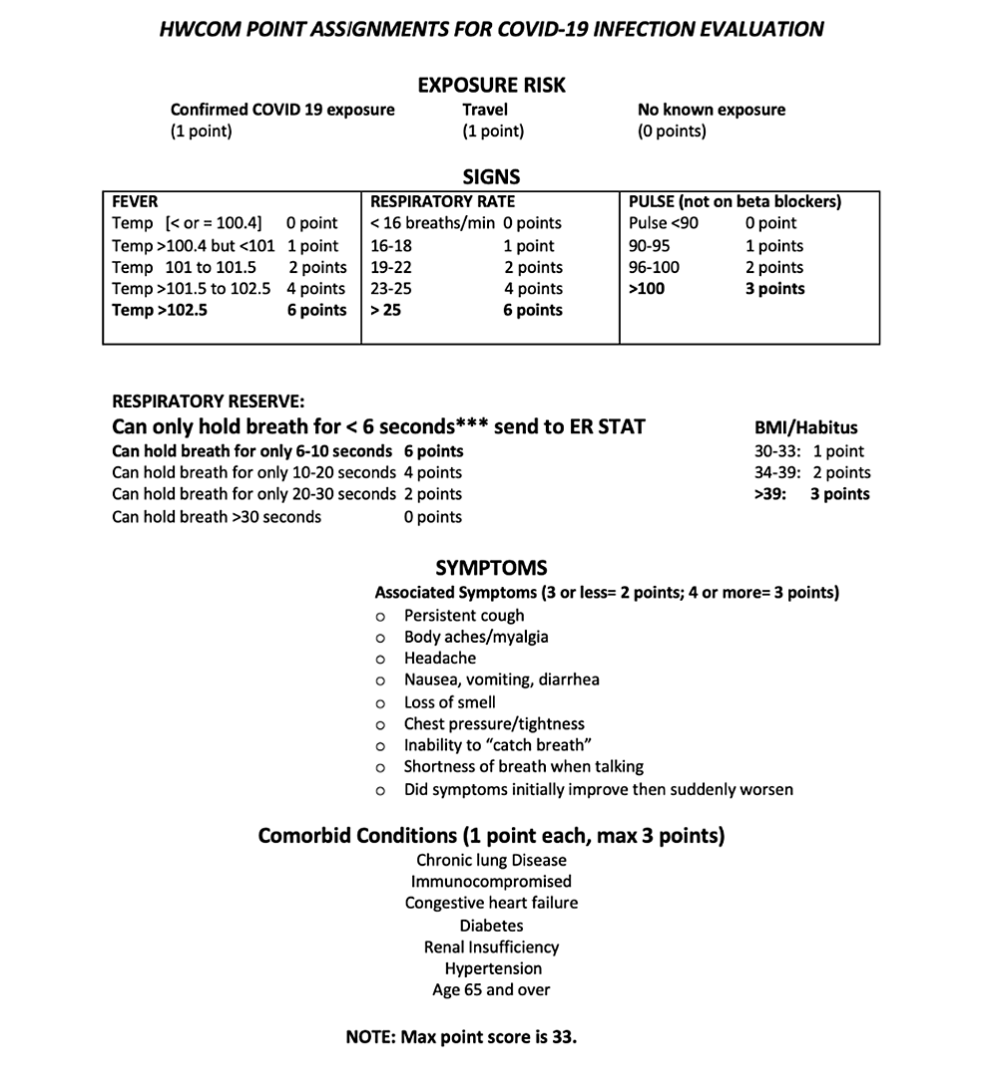
COVID-19 risk assessment tool used by medical students developed in conjunction with the faculty of HWCOM and the Miami-Dade County Homeless Trust

The legal team of the Homeless Trust, in collaboration with FIU faculty, formulated an official risk assessment tool and distributed it to all medical students and faculty to use as a template for their daily phone encounters. The risk assessment tool, as seen in Figure 1, assessed the risk of COVID-19 infection and severity based on clinical course and history of illness. Students then calculated a score based on symptoms and clinical history following the guide which would stratify patients into three categories: high, moderate, and low risk. The maximum score was 33. Low-risk (<16 points) patients would continue to be monitored daily. Moderate-risk patients (between 17 and 21 points) needed close follow-up over 48-72 hours. Patients were then provided a clinical appointment with contracted clinical staff from the Homeless Trust who visited the patients in person and performed further testing. High-risk patients (>22 points) were recommended to go to the Emergency Department for further evaluation. The Homeless Trust was contacted by the team for any moderate- or high-risk patients. After the phone encounter, the medical students were responsible for documenting the patient encounter within an electronic medical record (EMR).

Chart review process

**Figure 2:**
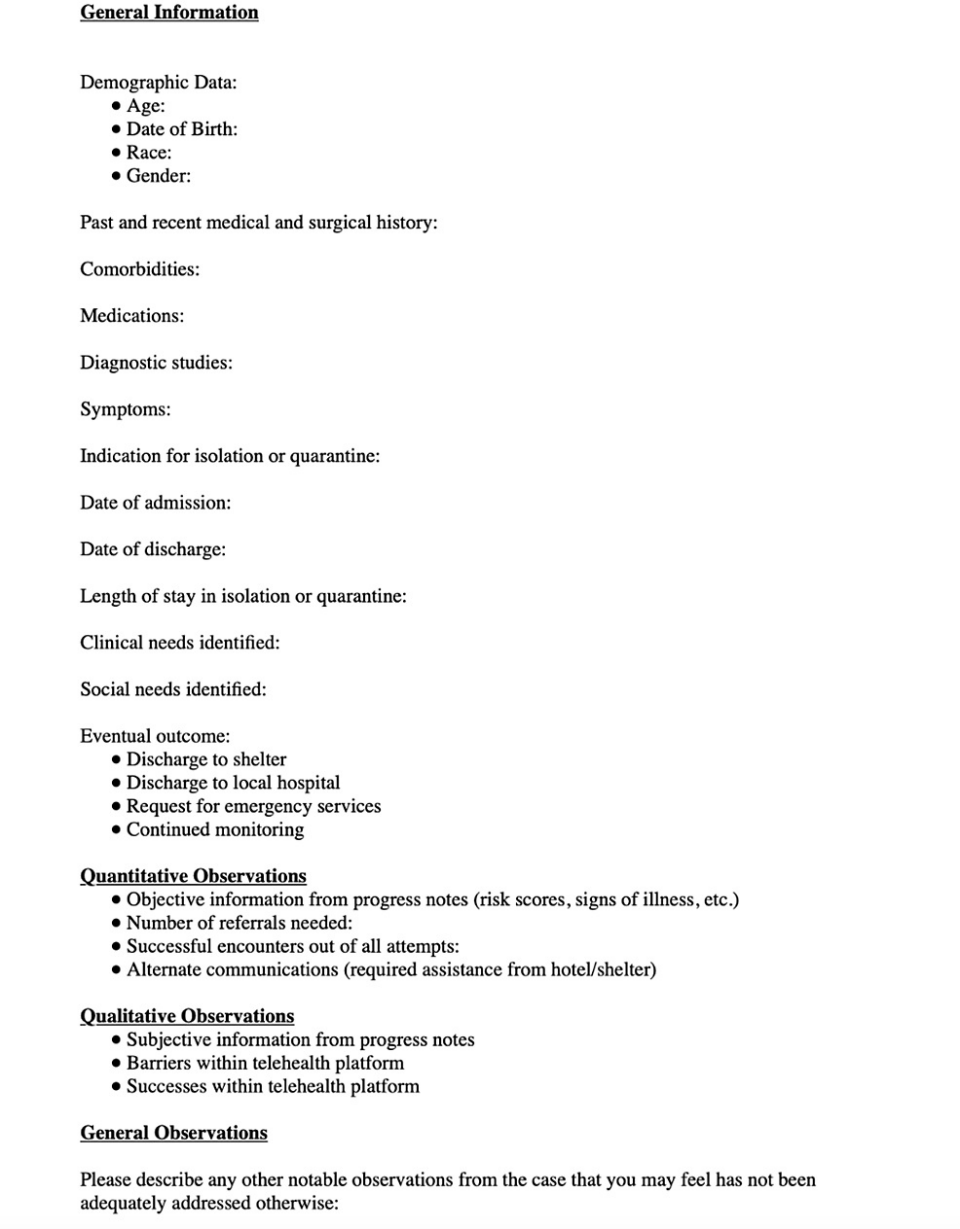
Standardized record review template utilized by medical student reviewers

A retrospective chart review of clinical documentation compiled from both medical students and faculty from the Centricity EMR was conducted using a standardized record review template approved by HWCOM faculty, as seen in Figure 2. Chart reviews were conducted by two of the investigators, KS and AS. Specific focus was given to both comorbidities, clinical needs, social needs, and qualitative data regarding barriers to care. Personal protected health information was systematically de-identified via masking personal identifiers as per our IRB protocol. Four exemplar cases were subsequently chosen by two medical students, VM and AM. Cases were selected to highlight the best strategies to utilize telemedicine among PEH while focusing on specific barriers to care that PEH face.

## Results

Demographics, length of stay, and frequency of contact

A total of 31 patients, between the ages of 20 and 84 and with an average age of 50.74 with a standard deviation of ± 5.986 and 95% CI (44.77-56.71), were followed in the program. There were eight females and 23 males in the study. Three patients identified as black/African American and 10 patients as Hispanic. Eighteen patients did not report their ethnicity/race. The average length of isolation for the group was 23.8 days with a range of 7-54 days. The average number of calls made per individual in quarantine or isolation was 13.45 phone calls with a range from 1 to 54 calls. The answer rate was approximately 72.4% with a total of 417 calls placed and 302 successful encounters. Ten patients had a 100% response rate; meaning that they picked up the phone with every call placed.

Medical comorbidities and chronic medications

The reported medical comorbidities in order of incidence are displayed in Table 1.

**Table 1:** Comorbidities reported by patients in order of incidence

Comorbidities	Incidence
Hypertension	45.16% (14 patients)
Type 2 diabetes mellitus	32.25% (10 patients)
Mental Illness (depression, schizophrenia, bipolar)	19.35% (6 patients)
Substance abuse	22.28% (7 patients)
HIV	6.45% (2 patients)
Asthma	6.45% (2 patients)
Gout	3.22% (1 patient)
Emphysema	3.22% (1 patient)
Pacemaker placement	3.22% (1 patient)
Dyslipidemia	3.22% (1 patient)
Hypothyroidism	3.22% (1 patient)

Table 2 highlights the medications that patients reported taking. 

**Table 2:** Medications that patients reported taking

Medications	Incidence
Blood pressure medication	29% (9 patients)
Metformin	25.8% (8 patients)
Insulin	12.9% (4 patients)
Psychiatric medication	12.9% (4 patients)
Albuterol	6.45% (2 patients)
HIV antivirals	6.45% (2 patients)
Lipid-lowering agents	3.22% (1 patient)
Thyroid medication	3.22% (1 patient)

COVID-19 positivity rate and COVID-19 risk assessment scores

Twenty-one of 31 patients (67.74%) tested positive for COVID-19. The remaining 10 patients met the criteria for quarantine due to exposure and/or symptoms. Using the formulated official risk assessment tool (see Figure 1), 29 patients had a score between 0 and 16 indicating a low COVID-19 risk score. Two patients had a score of 17, indicating a moderate-risk score. However, these two patients eventually scored below 16 points with subsequent encounters throughout their stay and were both placed in the low-risk category. No patient received a score higher than 22 points indicating a high-risk score.

Referrals for services and eventual disposition

The average number of referrals for services was 1.5 with a range of 0-4 referrals. Eleven patients were referred to the Homeless Trust or to the hosting hotel to access either toiletries, food items, clothes, new sheets, internet access, or acetaminophen. Four patients were referred to the Trust for medication access/refills including hypertension, diabetes, and dyslipidemia medications. Two patients requested urological consultations. Two patients requested psychiatric consultations. In terms of discharge disposition, 25 patients were discharged to the homeless shelter. Three patients required continued monitoring. Three patients were lost to follow-up due to a lack of communication toward the end of their stay.

Care-related themes

While each set of telehealth team encounters was unique, allowing the multi-disciplinary team to address each patient's individual needs, we found similar themes that highlight the strengths and challenges of providing telehealth services to PEH. These themes identified during the chart reviews centered around addressing: 1) the lack of basic necessities that PEH face; 2) the burden of chronic medical illnesses; 3) a lack of health literacy; 4) the burden of mental illnesses, and 5) the acute stress caused by COVID-19 itself.

There was a high burden of chronic diseases amongst the PEH we encountered, specifically hypertension and type 2 diabetes mellitus (24 patients total). These chronic diseases were often compounded by lower health literacy, with many patients unaware of the names of the medications they were taking, doses, or frequency. This initiative assisted with chronic disease management through a partnership of the Homeless Trust and the Camillus House, a large homeless shelter and treatment facility in Miami, which provided medication refills to several participants.

In addition, a large proportion of our patients carried previous or current diagnoses of mental health disorders. These disorders were complicated by the fact that many clients reported increased anxiety about their housing situation and the effects that the COVID-19 pandemic would have on it. After determining the individual risk score and finishing quarantine/isolation, many individuals were often discharged to a shelter, after which our monitoring stopped. Increased anxiety was also reported regarding the social isolation experienced during quarantine/isolation. Several individuals expressed that the regular contact provided by the telehealth encounters helped alleviate some of that social isolation. These common care-related themes are best highlighted by a few memorable encounters.

Exemplar patient 1

We highlight a 74-year-old Hispanic man with a history of unspecified heart disease and phantom leg syndrome who tested positive for COVID-19 and was admitted on 5/17/2020 and discharged on 05/22/2020. He answered every call that was placed. He scored 15 points on the initial risk assessment indicating a low risk. The patient required pain management, specifically acetaminophen, for his phantom limb pain following a car crash four years prior. The team was able to contact his primary care physician (PCP) for further patient history and coordinate with the onsite nurse practitioner to address his pain, eventually receiving acetaminophen. Social needs that were identified included a lack of understanding of the reason behind isolation and not being able to access belongings at the shelter following hasty seclusion for quarantine. The eventual outcome of this case was continued monitoring.

Exemplar patient 2

The case of a 58-year-old woman with a history of hypertension, type 2 diabetes mellitus, and depression further highlights the confluence of difficulties that our study population often faced and the efforts of our initiative to alleviate these difficulties. The patient struggled with access to medications, plus the ongoing burden of a mental health disorder combined with housing instability. She initially did not have any of her medications with her and had a subjective low mood with a low level of social support. Over the course of 23 successful phone calls, the student team was able to put in referrals to Camillus House to provide the patient with losartan, metformin, and insulin and to Behavioral Health at FIU where she was able to receive therapy. Throughout the duration of her isolation, she was COVID-negative with no symptoms and a corresponding low overall risk (score of three). Eventually, she was discharged to a shelter.

Exemplar patient 3

The case of a 75-year-old Hispanic man highlights the burden of chronic diseases combined with lower health literacy, housing instability, and the acute stress of COVID-19. He initially presented with a history of emphysema, hypertension, diabetes mellitus, tobacco abuse disorder, prostate and bladder cancer, arthritis, and urinary incontinence that required a catheter. It was unclear on presentation what anti-hypertensive and diabetic medications or chronic obstructive pulmonary disease (COPD) treatment he was receiving. He had tested positive for COVID-19 and had symptoms of shortness of breath and malaise with a low overall risk score of seven. Over the course of 51 successful encounters, the student team was able to place referrals for the individual to have a urology consult to help remove the catheter after he had difficulties with it. He had also endorsed anxiety about his living situation, so a referral was placed to the Homeless Trust to provide him with more housing information. When he had originally presented, he had difficulties acclimating to the telehealth platform, resulting in three unsuccessful outreach attempts. The hotel staff was able to help teach the individual to keep his phone charged to allow for successful communication. After a 54-day quarantine, the patient was discharged to a shelter.

Exemplar patient 4

The case of a 52-year-old African American man highlights the struggle to obtain basic necessities that PEH often face, the lack of accessible medical care, and the symptomatic burden of COVID-19 itself. He initially presented with a history of type 2 diabetes mellitus, a pacemaker in place for an unclear indication, and a recent hospitalization for pneumonia. He had run out of insulin after his discharge from the hospital, and his diabetes had been complicated by significant pain in his hands and feet which was concerning for diabetic neuropathy. The student health team was able to place a referral to Camillus House to have insulin delivered to the patient. Over the course of his 26-day stay, he had made several requests for toiletries and clothes; the student team was able to address these concerns by placing a referral to the hotel, which was able to deliver the necessities to the client. His symptoms of COVID-19 included cough, shortness of breath, a subjective fever, difficulty sleeping, and dizziness. Correspondingly he initially had a moderate risk score of 17 which over the course of his quarantine decreased to a low overall risk of 13. Eventually, the patient was discharged to a shelter.

## Discussion

The 31 individuals served by the HWCOM Telemedicine Homeless Monitoring Project illustrate crucial experiences that PEH faced during the pandemic. Using a risk assessment tool developed in collaboration with the Miami-Dade County Homeless Trust, we determined that for the majority of individuals within our cohort the burden of COVID-19 was low, as no high-risk patients were identified for the duration of our study. As the illustrating stories indicate, many of these individuals required extensive contact and multiple interactions with student health teams throughout the duration of their isolation or quarantine period to address both their social needs and access to basic medical care. The main areas addressed during these encounters include addressing the lack of basic necessities that PEH face, the burden of chronic illnesses, lack of health literacy, psychiatric comorbidities, and acute stress caused by COVID-19.

The average age of the 31 individuals included in our cohort was 50.7 years with an average length of isolation or quarantine of 23.8 days. The investigators hypothesize that the extended quarantine or isolation period, despite a relatively low COVID-19 disease burden, was due to the nature of homelessness itself. It was often difficult to find a safe place to discharge the clients. Intriguingly, our average length of stay involved a shorter isolation/quarantine period compared to that noted by Huggett and colleagues in Chicago, IL, who reported an average stay of 59 days, ranging from 18 to 115 days [7]. In our cohort, the overall rate of successful contact for the student health teams was 72.4%, with 302 successful encounters in total. As indicated above, using the COVID-19 risk assessment tool 93.5% of our cohort was considered low risk, with the remaining 6.5% in the moderate-risk category. Twenty-five out of 31 individuals were discharged to homeless shelters, while three individuals required continued monitoring. The remaining three individuals were lost to follow-up; giving the loss to follow-up rate to approximately 10%. This rate is similar to that reported by Huggett and colleagues who found that 26 out of 259 patients lost to follow-up (10%) [7]. This consistent, relatively low rate suggests that the PEH population appreciated this model of care.

As might be expected, PEH have difficulty accessing basic human needs. Around one-third of our patients requested a referral to either the Homeless Trust or the hosting hotel for access to basic supplies such as toiletries, food items, clothes, new sheets, internet access, or acetaminophen. Exemplar patient 4, who requested toiletries and clothes, which were later supplied by the hosting hotel, illustrates this phenomenon. This theme was underscored in a study by Riley and colleagues where they found that 66% of women experiencing homelessness had one or more subsistence requirements, including insufficient access to food, clothes, shelter, or hygiene supplies [8]. In a recent systematic review, the investigators highlight the theme of unmet basic human needs among PEH. They describe patterns of individuals experiencing financial struggles, food insecurity, poor nutrition, and difficulty managing daily life without laundry facilities or space for managing personal hygiene practices [2]. This lack of necessities is often overlooked by health and social care. Focusing on one’s medical health is difficult if basic needs are not met. A similar, separate systematic review by Corey and colleagues draws attention to how the pandemic affected aspects of people’s lives among the homeless population beyond the COVID-19 infection itself. They describe how the large-scale closure of public buildings and facilities reduced access to toilets and basic hygiene affecting unsheltered people. Additionally, due to the pandemic’s stay-at-home advisories, less financial support from the public and reorganization of food/social support services contributed to food insecurities in PEH [9].

Exemplar patients 2 and 3 in our case series showcase how PEH face a unique burden of chronic diseases. Out of our cohort of 31 homeless individuals, the most prevalent chronic diseases were hypertension (14 individuals) and diabetes mellitus (10 individuals). These individuals often struggled with regular access to medications which necessitated the student teams to place referrals to the Homeless Trust for medication refills (four individuals). The prevalence of hypertension and diabetes mellitus within our cohort is comparable to a 2021 study by Huggett and colleagues. Their study focused on the potential effects that protective housing interventions would have on PEH with chronic diseases during the COVID-19 pandemic [7]. They found that hypertensive and diabetic individuals had better blood pressure and glycemic controls with protective housing than without. Although our study primarily focused on providing COVID-19 risk assessment for PEH during a period of isolation or quarantine, student health teams often sent referrals for medication refills to individuals diagnosed with chronic diseases during the period of the study. Our study did not look at blood pressure or diabetes control as endpoints, but the impact that regular medication refills have on such control amongst PEH is an area that necessitates further study.

Health literacy was another theme noticed in this PEH sample population, illustrated by Exemplar patient 3. A few patients reported that they were unsure of what medications and/or what dosages they were taking, raising questions about their adherence to their treatment plan. Among low-income patients, low health literacy is associated with increased rates of hospitalization and poorer health outcomes [10], and we suspect that the patients in our cohort were at similar risk. Additionally, there was one patient in our sample group with a history of bipolar disorder and depression who did not take her prescribed medication because she did not believe in its efficacy. The pattern of nonadherence to treatment for psychiatric conditions is also seen in the Huggett study, where 146 out of 259 patients had mental health conditions while only half of them were being treated [7].

Six individuals within our cohort reported mental illness (depression, schizophrenia, bipolar disorder) as a prior diagnosis. Two individuals requested psychiatric consultations from the student health teams, requiring placing referrals. The burden of mental illness was highlighted by Exemplar patient 2. The prevalence of mental health disorders was similar in the cohort described in a 2021 study by Fuch and colleagues [5]. The study further highlighted that mental illnesses were not associated with a decreased length of isolation/quarantine. Our findings were similar, with an average length of stay of 23.8 days in all individuals in our cohort versus 23.3 days in individuals with mental health disorders. A 2022 systematic review by Corey and colleagues demonstrated that nine studies showed poorer mental health outcomes for PEH during the COVID-19 pandemic [9]. Our study did not assess the outcomes of mental health disorders amongst our cohort. A study by Garvin and colleagues, however, demonstrated increased use of telehealth mental health services amongst veterans experiencing homelessness [11]. The impact that our telemedicine platform had on the burden of mental illnesses amongst our cohort of PEH during the COVID-19 isolation/quarantine period is an area that requires further study.

Numerous individuals within our cohort reported increased anxiety due to the impact that the COVID-19 pandemic would have on their housing situation. Increased anxiety was also reported with regard to increased social isolation during the isolation/quarantine period. These phenomena are highlighted by Exemplar patients 2 and 3. Data compiled by van Ruth and colleagues from a survey of homeless individuals in Hamburg found that a diagnosis of anxiety/depression was the second most common variable that influenced the health-related quality of life amongst PEH [12]. Within our cohort, one individual stated the regular telehealth check-ins with the student health teams helped combat the feelings of isolation brought on by quarantine, highlighting the impact that our pilot study may have had on combating the increased stress PEH faced due to the COVID-19 pandemic. Further studies are needed to assess the effectiveness of similar models in regard to combating acute stress.

Several methodological limitations of the present study need to be acknowledged. The small population size of 31 patients limits generalizability to larger populations. Additionally, the nature of qualitative research in this case series also poses a limitation. While the screening risk tool used for this study was designed in collaboration with the Homeless Trust and FIU Faculty, the tool was not independently validated. Lastly, the 10% loss to follow-up rate and 72.4% answer rate may pose another limitation to this study. Areas of further exploration for future studies include exploring the impact of the program from the perspective of different stakeholders, including patients, the Homeless Trust, and the faculty and staff participants.

## Conclusions

For this cohort, the telehealth monitoring project proved to be a valuable service to both patients and the medical student-faculty teams involved. The monitoring program allowed PEH to isolate/quarantine without the need for a hospital stay, which relieved some of the burdens of the saturated hospital systems while supporting our local county infrastructure. The collaboration between the Homeless Trust and FIU HWCOM provided a valuable learning experience for medical students by allowing them to be involved in clinical care amidst the lockdown environment due to the COVID-19 pandemic. We suggest models such as these will prove fruitful for future pandemics.
